# Antioxidant, Osteogenic, and Neuroprotective Effects of Homotaurine in Aging and Parkinson’s Disease Models

**DOI:** 10.3390/antiox14030249

**Published:** 2025-02-21

**Authors:** Arianna Minoia, Francesca Cristiana Piritore, Silvia Bolognin, João Pessoa, Bruno Bernardes de Jesus, Natascia Tiso, Maria Grazia Romanelli, Jens Christian Schwamborn, Luca Dalle Carbonare, Maria Teresa Valenti

**Affiliations:** 1 Department of Engineering for the Innovation Medicine, University of Verona, 37100 Verona, Italy; arianna.minoia@univr.it (A.M.); luca.dallecarbonare@univr.it (L.D.C.); 2 Department of Neurosciences, Biomedicine and Movement Sciences, University of Verona, 37100 Verona, Italy; francescacristiana.piritore@univr.it (F.C.P.); mariagrazia.romanelli@univr.it (M.G.R.); 3 Luxembourg Centre for Systems Biomedicine (LCSB), Developmental and Cellular Biology, University of Luxembourg, L-4367 Belvaux, Luxembourg; silvia.bolognin@maastrichtuniversity.nl (S.B.); jens.schwamborn@uni.lu (J.C.S.); 4 Department of Medical Sciences and Institute of Biomedicine—iBiMED, University of Aveiro, 3810-193 Aveiro, Portugal; joao.pessoa@ua.pt (J.P.); brunob.jesus@ua.pt (B.B.d.J.); 5 Department of Biology, University of Padova, 35131 Padova, Italy; natascia.tiso@unipd.it

**Keywords:** homotaurine, reactive oxygen species, mesenchymal stem cells, Parkinson’s disease

## Abstract

Aging is associated with the accumulation of cellular damage due to oxidative stress and chronic low-grade inflammation, collectively referred to as “inflammaging”. This contributes to the functional decline in various tissues, including the brain and skeletal system, which closely interplay. Mesenchymal stem cells (MSCs), known for their regenerative potential and ability to modulate inflammation, offer a promising therapeutic approach to counteract aging-related declines. In this study, we investigated the effects of homotaurine (a small molecule with neuroprotective properties) on MSCs and its effects on osteogenesis. We found that homotaurine treatment significantly reduced reactive oxygen species (ROS) levels, improved MSC viability, and modulated key stress response pathways, including the sestrin 1 and p21 proteins. Furthermore, homotaurine promoted osteogenesis and angiogenesis in zebrafish models by enhancing the expression of critical osteogenesis-associated genes, such as those coding for β-catenin and Runt-related transcription factor 2 (Runx2), and increasing the levels of the kinase insert domain receptor-like angiogenesis marker in aged zebrafish. In Parkinson’s disease models using patient-specific midbrain organoids with the leucine-rich repeat kinase 2 G2019S mutation, homotaurine treatment enhanced β-catenin expression and reduced ROS levels, highlighting its potential to counteract the oxidative stress and dysfunctional signaling pathways associated with neurodegeneration. Our findings suggest that homotaurine not only offers neuroprotective benefits but also holds promise as a dual-target therapeutic strategy for enhancing both neuronal and bone homeostasis in aging and neurodegenerative diseases.

## 1. Introduction

During aging, cells accumulate damage from oxidative stress and inflammation, a phenomenon often referred to as “inflammaging” [1,2,3]. This chronic low-grade inflammation contributes to the functional decline in tissues and organs, including the brain and the skeletal system. Mesenchymal stem cells (MSCs), found in the bone marrow, fat, and other tissues, offer a promising approach to counteracting this decline. MSCs have the ability to differentiate into various cell types, including bone, cartilage, and fat cells. More importantly, even when they do not differentiate into new cells, MSCs release signaling molecules that promote tissue repair, reduce inflammation, and stimulate the body’s intrinsic regenerative processes [4,5,6].

However, MSCs are not protected from the effects of aging. Over time, these cells gradually lose their regenerative capacity, a challenge that ongoing studies are working to overcome. Strategies including harvesting younger MSCs from sources such as umbilical cord tissue or reprogramming them to restore youthful properties are currently being explored [7]. In the context of aging, MSCs hold particular promise for the treatment of conditions like osteoporosis, a disease characterized by bone loss and fragility, which becomes more common with age [8]. Notably, osteoporosis is often associated with neurodegenerative diseases, including Parkinson’s disease [9,10,11,12]. This suggests that aging, chronic inflammation, and metabolic alterations may create a shared biological environment that impacts both the skeletal and nervous systems. Understanding how these systems influence each other could lead to integrated therapeutic strategies that target both bone loss and neurodegeneration.

Homotaurine, a small organic molecule analogue of taurine, has gained attention for its potential neuroprotective properties. It was initially studied in the context of Alzheimer’s disease because of its ability to prevent the aggregation of β-amyloid peptides, one of the hallmarks of this degenerative condition [13,14].

Similarly to the brain, the skeletal system undergoes significant changes during aging, and an intriguing link has been observed between the decline in skeletal health and the incidence of neurodegenerative diseases such as Parkinson’s diseases [15]. Patients with Parkinson’s diseases have an increased risk of osteoporosis and fractures, a vulnerability influenced by reduced mobility and the bone-loss-accelerating side effects of certain medications used to manage the disease [16]. Bone aging, coupled with the reduction in the regenerative capacity of MSCs, is the primary risk factor for osteoporosis—a condition characterized by a decline in bone mineral density that substantially increases fracture susceptibility in the elderly population [8].

Given these interconnections between aging, bone loss, and neurodegeneration, our study aimed to investigate the effects of homotaurine on MSCs, with a particular focus on its potential to counteract the aging phenotype. To the best of our knowledge, this is the first study to explore homotaurine’s effects on the osteogenic differentiation potential of MSCs and its impact on a 3D Parkinson’s disease organoid model. While previous studies have largely focused on homotaurine’s neuroprotective properties, our research uniquely examines its dual role in promoting both skeletal regeneration and neuroprotection. This approach is important because it reveals how homotaurine might not only protect neurons in diseases like Parkinson’s but also support bone health by enhancing MSC quality and osteogenesis. By combining these two aspects, we aim to provide a proof-of-concept study on new therapeutic possibilities that could bridge the gap between skeletal health and neurodegeneration, addressing an area that has remained largely unexplored until now.

## 2. Material and Methods

### 2.1. Cell Cultures

Human mesenchymal stem cells (hMSCs, PromoCell, Heidelberg, Germany), were plated at a density of 3.0 × 10^5^ cells in T75 flasks and cultured in the presence of mesenchymal stem cell growth medium (Cat. number 12746012, Gibco, Thermo Fisher Corporation, Waltham, MA, USA). Cells were incubated at 37 °C in a humidified atmosphere with 5% CO_2_. The culture medium was completed with antibiotics (1% penicillin/streptomycin/amphotericin B, Cat. number 17-745E Lonza—Walkersville, MD, USA) and 1% L-glutamine (Cat. number G7513, Merck—Darmstadt, Germany).

At 70% confluence, cells were mock-treated or treated with homotaurine (lot: 200029216, code: 2200000006; Laborest S.r.l., Assago, MI, Italy), as specified in the following sections. The concentrations of homotaurine were selected based on the data available in the literature [17,18].

### 2.2. CCK-8 Assay

Cell viability was assessed using a Cell Counting Kit-8 (CCK-8, IK-11133-3000; Immunological Sciences, Rome, Italy). For the CCK-8 assay, cells were seeded in 96-well plates at a density of 3.0 × 10^4^ cells per well. Cells were cultured in the presence of mesenchymal stem cell growth medium in the absence or presence of 100 µM homotaurine. At each time point, the cell viability was assessed using a CCK-8 Assay Kit. Briefly, after a wash with PBS (Cat. number SH30256.0; Cytiva, Washington, DC, USA), 100 µL of mesenchymal stem cell growth medium with 10% CCK-8 solution were added to each well, and the plate was incubated for 2 h at 37 °C. The absorbance at 450 nm was then measured using the microplate reader Tecan Infinite M200 (Tecan, Männedorf, Switzerland).

### 2.3. Zebrafish

Zebrafish experiments were conducted at the Interdepartmental Centre of Experimental Research Service (CIRSAL), University of Verona, Italy, in compliance with the approved experimental protocol authorized by the Directorate-General for Animal Health and Veterinary Medicinal Products, Italian Ministry of Health (authorization code number 447/2023-PR). The fish were randomly selected based on their age, and the sample size was determined to ensure the achievement of statistical significance in the results. Suffering animals were excluded from this study. Embryos were derived from KDRL-GFP adults expressing green fluorescence protein (GFP) under the promoter of the KDR (kinase insert domain receptor) gene following standard methodologies [19,20] and cultured at 33 °C in water supplemented with 0.25 mM homotaurine starting from 2 days post-fertilization (2 dpf) (control number: 90; treated number 90). Homotaurine supplementation was continued for 14 days, corresponding to the final experimental time point (16 dpf). Adult (4 months old; control number: 7; treated number: 8) and aged (36 months old; control number: 3; treated number: 3) zebrafish were maintained in water containing either 0.25 mM homotaurine or untreated control water for 7 days. After the treatment period, zebrafish were euthanized and collected for molecular analyses, as detailed in the subsequent sections. Fluorescence imaging was conducted using a Leica M205FA fluorescence microscope (Leica Microsystems, Wetzlar, Germany), and fluorescently stained areas were quantified using ImageJ software version 1.54m, as described previously [21].

### 2.4. Generation of Midbrain Organoids

Neuronal epithelial stem cells (NESCs) were derived from human-induced pluripotent stem cells (iPSCs) (A13777, Gibco-ThermoFisher Corporation, Waltham, MA, USA), as described by Reinhardt et al. [22]. These NESCs, which carry the leucine-rich repeat kinase 2 (LRRK2) G2019S mutation, served as the starting population for midbrain organoid generation, following the protocol established by Zagare et al. [23]. Briefly, NESCs were maintained on Geltrex-coated 6-well plates and routinely passaged at 80–90% confluence. At this stage, cells were detached with accutase (A6964-500M, Sigma-Aldrich, St. Louis, MO, USA), and viable cells were counted using the Trypan Blue (T10282, Invitrogen-ThermoFisher Corporation, Waltham, MA, USA) exclusion method with an automated cell counter (Countess™ II, Invitrogen-ThermoFisher Corporation, Waltham, MA, USA). For 3D culture initiation, 9 × 10^5^ cells were suspended in 15 mL of N2B27 maintenance medium [23], and 150 µL of this suspension was seeded into each well of a 96-well ultra-low attachment (ULA) plate (7007, Corning-ThermoFisher Corporation, Waltham, MA, USA). Medium changes were performed every other day. On day 2, the N2B27 maintenance medium was replaced with N2B27 patterning medium [23], which was subsequently substituted with N2B27 differentiation medium [23] on day 8. At this point, treatment with homotaurine at a final concentration of 0.25 mM was initiated. Long-term cultures underwent full medium changes every 3–4 days using 500 µL of N2B27 differentiation medium. The 3D cultures were cultured for 40 days. Non-embedded organoids were collected into Eppendorf tubes and stored at −80 °C for RNA extraction, as described below. On day 8, eight 3D colonies were transferred to an untreated 24-well tissue culture plate (TCP), where each well was filled with 500 µL of pre-warmed (37 °C) N2B27 maintenance medium. Each colony was embedded in Geltrex droplets (30 µL per colony), ensuring the colony was centrally positioned. The Geltrex was allowed to polymerize for 25 min at 37 °C, after which the medium was replaced with N2B27 differentiation medium, and the compound treatment was initiated. Embedded organoids were cultured for 40 days and subsequently processed for sectioning and staining, whereas non-embedded organoids were utilized for total RNA extraction using Qiagen mRNA extraction and protein extraction kits (Qiagen, Milan, Italy), as described below.

### 2.5. Midbrain Organoid Size Measurement

To quantify the size of the midbrain organoids during differentiation, brightfield images were acquired for each cell line and culture condition using a stereomicroscope SMZ25 (Nikon, Minato-Tokyo, Japan) and analyzed using NIS imaging software version 6.10.01 (Nikon, Minato-Tokyo, Japan). Colony area measurements were conducted to assess the morphological changes over time.

### 2.6. Western Blotting

Proteins were extracted using RIPA buffer (Cat. no. 89900, Thermo Fisher Scientific, Waltham, MA, USA) according to the manufacturer’s instructions. Concentrations were determined using a BCA assay (Thermo Scientific, Waltham, MA, USA). Protein samples were separated using sodium dodecyl sulfate-polyacrylamide gel electrophoresis (SDS PAGE) and then transferred to PVDF membranes (Thermo Fisher Scientific, Waltham, MA, USA). The PVDF membranes were probed with primary β-actin (MA1-140; Invitrogen, Waltham, MA, USA); SESN1 (PA5-98142; Invitrogen, Waltham, MA, USA); SESN2 (ab-178518; Abcam, Cambridge, MA, USA); p53 (ab 06083; FineTest, Hubei, China); p21 (A2691; ABclonal, Düsseldorf, Germany); β-catenin (PA5-19469; Invitrogen, Waltham, MA, USA); P-β-catenin (5615; Cell Signaling, Danvers, MA, USA); RUNX 2 (71042; Cell Signaling, Danvers, MA, USA); and secondary antibodies (Anti-mouse (7076, Cell Signaling, Danvers, MA, USA); Anti-rabbit (7074, Cell Signaling, Danvers, MA, USA)), as previously reported [24].

### 2.7. Total RNA Extraction and Reverse Transcription (RT)

Total RNA extraction and reverse transcription (RT) were conducted following previously described protocols [4]. In brief, cells were collected, and total RNA was extracted using an RNeasy^®^ Protect Mini Kit (Qiagen, Hilden, Germany) in accordance with the manufacturer’s instructions. RNA concentration and quality were assessed using a Qubit™ RNA HS Assay Kit (Invitrogen, Waltham, MA, USA) and a Qubit 3 Fluorometer (Thermo Fisher Scientific, Waltham, MA, USA; catalog number: Q3321). Reverse transcription was performed using a First-Strand cDNA Synthesis Kit (GE Healthcare, Chicago, IL, USA) to synthesize complementary DNA (cDNA) from the isolated RNA. The resulting cDNA was utilized as a template for subsequent real-time quantitative PCR (RT-qPCR) analysis.

### 2.8. Real-Time Quantitative PCR

#### 2.8.1. TaqMan Real-Time PCR

Real-time quantitative PCR (RT-qPCR) was performed to assess gene expression levels. Each PCR reaction was carried out in a total volume of 20 µL, comprising 2 µL of cDNA, PCR master mix containing carboxyl-X-rhodamine (ROX), predesigned gene-specific primers, and probe sets. These primers and probes were sourced from the assay-on-demand gene expression products (Thermo Fisher Scientific, Waltham, MA, USA) and targeted *CTNNB1* (Hs00355045_m19) and *β-actin* (Hs99999903_m1).

#### 2.8.2. SYBR Green Real-Time PCR

Real-time quantitative PCR (RT-qPCR) was performed in a total reaction volume of 20 μL, comprising PCR Master Mix with ROX premixed with SYBR Green and 20 ng of cDNA from each sample. The following custom primer sets (Invitrogen, Carlsbad, CA, USA) were employed: *runx2a* (fw: GACGGTGGTGACGGTAATGG, rv TGCGGTGGGTTCGTGAATA), *runx2b* (fw CGGCTCCTACCAGTTCTCCA, rv CCATCTCCCTCCACTCCTCC), *actb1* (fw: CAGCCTTCCTTCCTGGGTATG, rw: ATGTCCACGTCGCACTTCAT), *ctnnb1* (Dr.) (fw: ATTGTGGAGGCTGGTGGC, rw: CCCTCCTGTTTGGTGGCG), and *sp7* (fw: GGCTATGCTAACYGCGACCTG, rw: GGCTATGCTAACTGCGACCTG).

The thermal cycling conditions included an initial denaturation step, followed by 45 amplification cycles, as previously reported [25].

The fluorescence signal was captured and analyzed using a real-time PCR system. For each RNA sample, three independent experiments were conducted, each with three technical replicates. Gene expression levels were quantified using the ΔΔCt method, as calculated with TaqMan SDS analysis software version 2.3 (Thermo Fisher Scientific, Waltham, MA, USA), in accordance with previously described methods [26].

### 2.9. Confocal Analysis of NESCs 3D Colonies

NESCs 3D colonies were fixed in 4% paraformaldehyde (PFA; P6148, Sigma-Aldrich, St. Louis, MO, USA), embedded in 3% agarose, and sectioned into 70 µm slices using a Vibratome (Leica, Wetzlar, Germany). The sections were collected into 24-well plates containing PBS (Gibco, Thermo Fisher Scientific, Waltham, MA, USA). For immunostaining, the sections were permeabilized with 0.5% Triton X-100 (Sigma-Aldrich, St. Louis, MO, USA) in PBS for 30 min at room temperature (RT) on a shaker, followed by two washes with 0.01% Triton X-100 in PBS for 5 min each at RT on a shaker. Blocking was performed using a blocking buffer for 2 h at RT on a shaker. Primary antibody incubation (Table 1) was carried out for 48 h at RT. The sections were then washed three times with 0.01% Triton X-100 in PBS for 10 min each at RT on a shaker. Secondary antibody incubation (dilution 1:1000; Table 1) was performed in blocking buffer for 2 h at RT on a shaker. Subsequently, the sections were washed three times with 0.01% Triton X-100 in PBS for 10 min each at RT on a shaker, followed by a final wash in distilled water. Sections were collected with a brush and placed on object slides with a grid. To prevent sections from floating away, they were allowed to dry slightly before mounting and covering with a cover glass. Confocal microscopy (Leica, Wetzlar, Germany) was used to visualize three neuronal markers, and fluorescently stained areas (three areas in triplicate) were quantified in a semi-quantitative manner using ImageJ software, as previously reported [27].

### 2.10. ROS Detection

For the detection of reactive oxygen species (ROS) levels, distinct experimental protocols were employed for MSCs and NESC organoids.

In the case of MSCs, cells were seeded in 6-well plates at a density of 30.0 × 10^4^ cells per well and cultured using mesenchymal stem cell growth medium (PromoCell, Heidelberg, Germany), with or without homotaurine at a concentration of 100 µM. The experiment was conducted at four different time points: 24 h, 3 days, 7 days, and 14 days. ROS levels were assessed using CellROX™ Deep Red Reagent (Cat. Number C10422; Invitrogen, Waltham, MA, USA), following the manufacturer’s protocol. At each time point, cells were washed in PBS, detached, and counted before being incubated with 2 µM CellROX Deep Red Reagent for 30 min at 37 °C. Following incubation, cells were washed in PBS and analyzed using a BD Accuri™ C6 flow cytometer (BD Biosciences, Franklin Lakes, NJ, USA) to measure fluorescence intensity. Data collected at each time point were analyzed to determine ROS production.

For analyses of ROS in organoids, 8 samples for each condition were pooled. Organoid cells were detached by incubating them with Accutase (A6964-500M, Sigma, St. Louis, MO, USA) for 20–30 min to enable the entire network to detach as a single structure. Gentle pipetting was then applied, followed by an additional 5 min incubation at 37 °C to ensure complete disaggregation. Detached cells were pelleted by centrifugation at 400× *g* for 5 min in 1.5 mL tubes and washed twice with 1% BSA in PBS. For ROS detection, cells were incubated with 5 μg/mL 2,7-dichlorofluorescein diacetate (DCFDA) (D6883, Sigma, St. Louis, MO, USA) in fresh medium for 30 min.

Subsequently, cells were transferred into FACS tubes, kept on ice, and analyzed using a BD LSRFortessa™ X-20 Cell Analyzer (BD Biosciences, Franklin Lakes, NJ, USA). During analysis, dead cells were excluded based on propidium iodide (PI) staining (Cat. number: BMS500PI, eBioscience ThermoFisher, Waltham, MA, USA). Untreated control cells were included to assess background fluorescence, and data from cytofluorimetric imaging were analyzed using FlowJo software v10 (BD Biosciences, Franklin Lakes, NJ, USA).

### 2.11. Statistical Analysis

The statistical analysis was carried out using SPSS for Windows, version 22.0 (SPSS Inc., Chicago, IL, USA). Experimental data are presented as mean ± standard deviation (SD). To assess the significance of the differences between control groups and the corresponding experimental conditions, a two-tailed paired Student’s t-test was applied. Statistical significance was defined as a *p*-value < 0.05. For in vitro experiments, data were analyzed from a minimum of three independent replicates.

## 3. Results

### 3.1. Effects of Homotaurine on ROS Levels, Cell Viability, and Levels of Sestrin, p53, and p21 in Aging Mesenchymal Stem Cells

**Figure 1:**
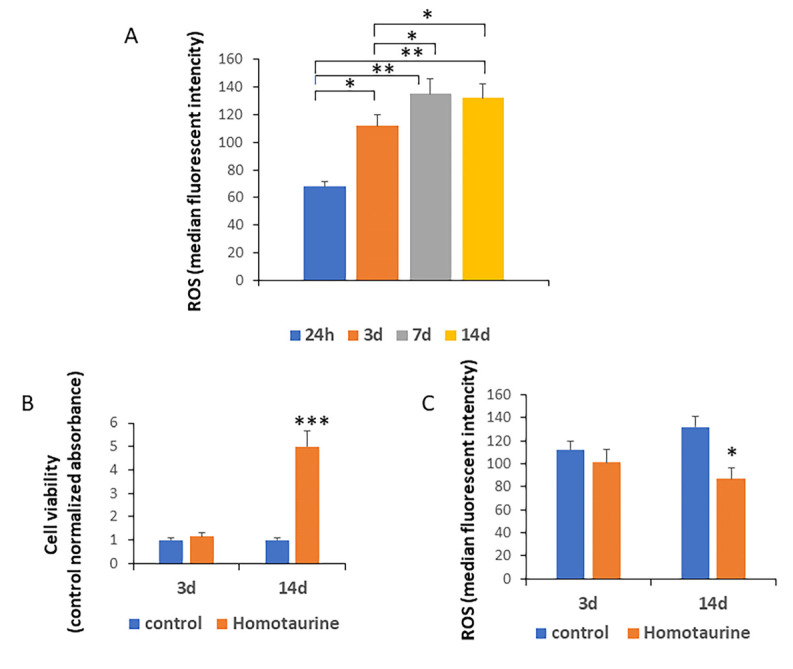
Assessment of ROS levels, MSC viability, and the effect of homotaurine treatment during a 14-day period. (**A**) A significant increase in reactive oxygen species (ROS) levels was observed starting from day 3, suggesting a correlation between elevated ROS and cellular aging in mesenchymal stem cells (MSCs). (**B**) No significant differences in cell viability were noted after 3 days of culture, while a marked improvement in cell viability was observed on day 14. (**C**) This cell viability increase was associated with a reduction in ROS levels following homotaurine treatment, also on day 14, with no previous alteration after 3 days of treatment. (* *p* < 0.05; ** *p* < 0.01; *** *p* < 0.005).

Since MSCs are critically affected by the aging process, we first sought to investigate whether ROS levels increase in MSCs cultured over extended time periods, as ROS are known to accumulate during aging. As shown in Figure 1A, we observed a significant increase in ROS levels, starting at the third day of culture and becoming even higher after day 7, which suggests a correlation between elevated ROS levels and the progression of cellular aging in MSCs.

We then aimed to assess whether homotaurine could influence the viability of MSCs. Our observations revealed that homotaurine treatment had a positive effect on cell viability on day 14 of culture compared to untreated cells, whereas no differences were observed after only 3 days of culture (Figure 1B). Interestingly, the improvement in cell viability observed on day 14 was associated with a reduction in ROS levels under the homotaurine treatment conditions (Figure 1C).

**Figure 2:**
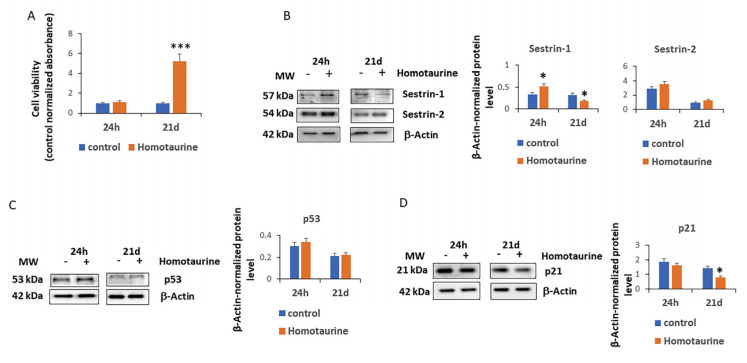
Effect of homotaurine on cell viability, sestrin expression, and cell cycle regulators in MSCs at 24 h and 21 days of treatment. (**A**) The positive effects of homotaurine on cell viability were observed after 21 days of treatment. (**B**) A significant increase in sestrin 1 levels was detected after 24 h of treatment; however, a decrease was detected at 21 days of homotaurine treatment, while sestrin 2 levels remained unchanged. (**C**) p53 levels did not show any significant changes in homotaurine-treated cells, while (**D**) a significant reduction in p21 levels was observed after 21 days of treatment. (* *p* < 0.05; *** *p* < 0.005). The original Western blot images are provided in the Supplementary Materials S1 and S2.

The positive effects of homotaurine on cell viability were also observed after 21 days of treatment, when homotaurine treatment increased cell viability (Figure 2A).

Given the critical role of sestrins in regulating cellular stress responses and maintaining cellular homeostasis [28], we analyzed the levels of sestrins 1 and 2 in MSCs treated with homotaurine. Our results revealed a significant increase in sestrin 1 levels after 24 h; however, after 21 days of treatment, there was a reduction in its levels (Figure 2B). In contrast, sestrin 2 levels did not show any significant changes at either of the two time points (Figure 2B). These findings highlight a specific modulation of sestrin 1 in response to homotaurine treatment, suggesting its potential involvement in the observed cellular benefits. We then analyzed the levels of p53 and p21 in homotaurine-treated cells, as both proteins are well-known regulators of the cell cycle and stress responses [29,30]. Our observations revealed that p53 levels remained unchanged in treated cells compared to the respective controls (Figure 2C). However, a significant reduction in p21 levels was observed in MSCs treated with homotaurine for 21 days (Figure 2D). These findings suggest a selective modulation of sestrin 1 and p21 in response to homotaurine, correlated with increased cell viability, with potential implications for stress response and cellular homeostasis.

### 3.2. Homotaurine Promotes Osteogenesis-Associated Gene Expression and Enhances Angiogenesis in Zebrafish Model

**Figure 3:**
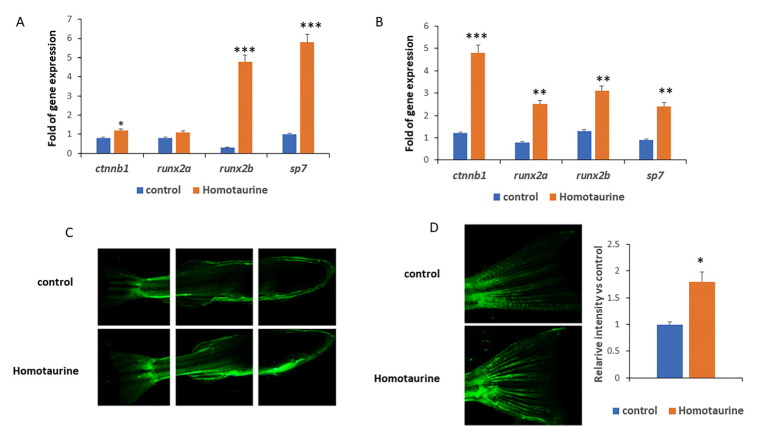
Effects of homotaurine on osteogenesis and angiogenesis gene expression in zebrafish. (**A**) In zebrafish larvae treated for 14 days, homotaurine significantly increased the expressions of the *ctnnb1*, *runx2b*, and *sp7* genes, crucial for skeletal formation. (**B**) Similar effects on the skeletal gene expression levels of *ctnnb1*, *runx2b*, *runx2b*, and *sp7* were observed in 4-month-old zebrafish. (**C**,**D**) In aged zebrafish (36 months old) treated with homotaurine for 7 days, (**C**) vascular endothelial growth factor receptor 2 (VEGFR2) expression was notably enhanced, (**D**) particularly in the tail region, as visualized in the kinase insert domain receptor-like–enhanced green fluorescent protein (KDRL-EGFP) transgenic model. Magnifications 4x (**C**) and 10x (**D**); (* *p* < 0.05; ** *p* < 0.01; *** *p* < 0.005).

Given the critical role of stem cells in skeleton maintenance, we sought to investigate the effects of 14-day treatments with homotaurine on the expression of the genes associated with skeletal development in zebrafish (*Danio rerio*) larvae. Our results revealed a significant increase in the expression of the *ctnnb1* (which codes for β-catenin), *Runx2b*, and *Sp7* genes, which are key regulators of osteogenesis and skeletal formation (Figure 3A). In addition, the effect of homotaurine on the expression of osteogenesis-associated genes *ctnnb1*, *runx2a*, *runx2b*, and *sp7* was also observed in 4-month-old zebrafish (Figure 3B), suggesting a sustained effect on skeletal development and maintenance.

It is well known that, during aging, angiogenesis processes are often impaired, leading to reduced vascular repair and regeneration [31]. This decline in angiogenic capacity is associated with various age-related diseases and represents a critical factor contributing to the overall aging process.

To investigate the effect of homotaurine on angiogenesis in an aged model, we used the transgenic KDRL-EGFP zebrafish line. The KDRL-EGFP (kinase insert domain receptor-like–enhanced green fluorescent protein) model is widely utilized in zebrafish research to study angiogenesis and vascular development. In this transgenic model, the promoter of the *kdrl* gene, which encodes a receptor analogous to human vascular endothelial growth factor receptor 2 (VEGFR2), fuses to EGFP to enable its fluorescent visualization in living animals.

As a result, the EGFP fluorescence intensity is directly proportional to the expression levels of the *kdrl* gene, allowing for the in vivo visualization of blood vessel formation and vascular patterning during development or in response to experimental treatments. For this study, we used a 36-month-old zebrafish model to investigate the effects of aging on angiogenesis. The aged fish were treated with 0.5 mM homotaurine for 7 days. Our observations revealed a significant increase in VEGFR2 expression (Figure 3C), particularly localized in the tail region (Figure 3D). These findings indicate that the homotaurine treatment promoted angiogenesis and enhanced the expression of osteogenesis-related genes in zebrafish at different development stages.

### 3.3. Homotaurine Decreases ROS Production and Upregulates RUNX2 and β-Catenin in a Parkinson’s Disease Organoid Model

**Figure 4:**
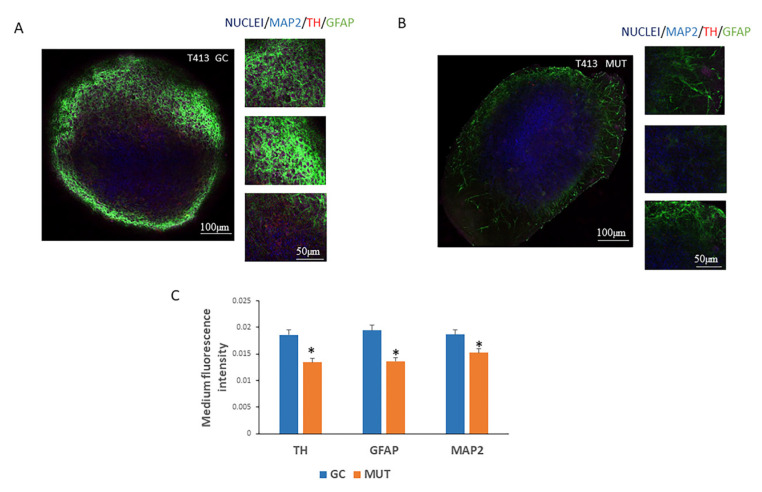
Expression of neuronal markers in 3D neuronal epithelial stem cell (NESC) organoids derived from induced pluripotent stem cells (iPSCs) carrying the leucine-rich repeat kinase 2 (LRRK2) G2019S mutation. (**A**) Three-dimensional NESC organoids generated from both gene-corrected (GC) and (**B**) mutated (MUT T413) iPSC lines were stained with antibodies against TH, GFAP, and MAP2 neuronal markers and visualized using confocal microscopy. Representative micrographs are shown. (**C**) Through a semi-quantitative analysis of the images, we observed reductions in the expression levels of all examined neuronal markers: tyrosine hydroxylase (TH), glial fibrillary acidic protein (GFAP), and microtubule-associated protein 2 (MAP2) in the NESC 3D colonies derived from the LRRK2-G2019S-mutated T413 cell line, compared to the gene-corrected GC counterpart. In blue nuclei, in purple the MAP2 marker, in red the TH marker and in green the GFAP marker (* *p* < 0.05).

Parkinson’s disease is a neurodegenerative disorder associated with aging, marked by the progressive loss of dopamine-producing neurons in the substantia nigra of the brain [32]. In this study, we investigated the effects of homotaurine in 3D NESC organoid colonies affected by the LRRK2-G2019S mutation, a key mutation associated with Parkinson’s disease. In particular, we used this cellular model to closely mimic the disease pathology and assess the potential impact of homotaurine treatment. We conducted an analysis using a confocal microscope to examine the expression of three neuronal markers that are generally downregulated in Parkinson’s disease patients, glial fibrillary acidic protein (GFAP), tyrosine hydroxylase (TH), and microtubule-associated protein 2 (MAP2). GFAP is an intermediate filament-III protein exclusively found in the astrocytes within the central nervous system, non-myelinating Schwann cells in the peripheral nervous system, and enteric glial cells. The expression of GFAP mRNA is subject to modulation by various factors, including nuclear-receptor hormones, growth factors, and lipopolysaccharides [33]. TH serves as a marker for neurons and endocrine cells containing dopamine, norepinephrine, and epinephrine (catecholamine). It is expressed transiently during development in neurons and neuroendocrine cells, which, in adulthood, either cease to express TH or express it at very low levels [34]. MAP2 plays a crucial role in the growth, differentiation, and plasticity of neurons. It has key functions in neuronal responses to growth factors, neurotransmitters, synaptic activity, and neurotoxins [35]. This study was carried out in patient-specific midbrain organoids derived from iPSCs with the LRRK2-G2019S mutation (MUT). As the control, the same iPSCs after gene correction into the wildtype version were used (GC) [22]. Thus, these three markers were fluorescently labeled and visualized by confocal microscopy in the organoids generated from both the GC and MUT T413 cell lines (Figure 4A,B). Four organoids were analyzed at 40 days of differentiation for each condition.

The semi-quantitative analysis revealed reductions in the expression levels of all three examined neuronal markers in the NESC 3D organoid colonies derived from the mutated T413 cell line compared to those from the gene-corrected counterpart (Figure 4C).

**Figure 5:**
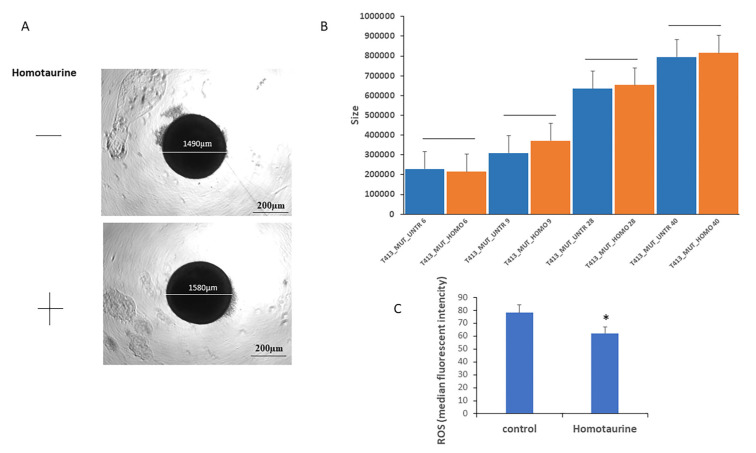
Evaluation of homotaurine’s effects on LRRK2-G2019S mutated midbrain organoids size and ROS levels during differentiation in 8 pooled organoids for each condition. (**A**) Representative images of mock-treated (top) and homotaurine-treated (bottom) NESC colonies, whose diameters are indicated. (**B**) The area (size, µm^2^) of LRRK2-G2019S-mutated midbrain organoids derived from mutated iPSC lines was assessed at multiple differentiation time points (6, 9, 28, and 40 days). Homotaurine treatment did not alter colony size compared to untreated (UNTR) controls, indicating that homotaurine does not adversely affect colony growth during differentiation. (**C**) Cytofluorimetric analysis revealed a significant reduction in ROS levels in homotaurine-treated samples at day 40 of differentiation. (* *p* < 0.05).

We then treated the organoids derived from the mutant cells with or without homotaurine to evaluate its effects. To assess the potential effects and toxicity of homotaurine, we conducted a detailed analysis of the dimensions of eight organoids at multiple differentiation time points (6, 9, 28, and 40 days). The results demonstrated that the homotaurine-treated colonies retained comparable dimensions to the untreated (UNTR) colonies, as shown in Figure 5A,B, suggesting that homotaurine does not adversely affect colony size during differentiation.

Furthermore, the beneficial effects of homotaurine were corroborated through cytofluorimetric analyses of ROS. Specifically, a significant reduction in ROS levels was observed in the homotaurine-treated samples compared to the untreated controls (Figure 5C).

**Figure 6:**
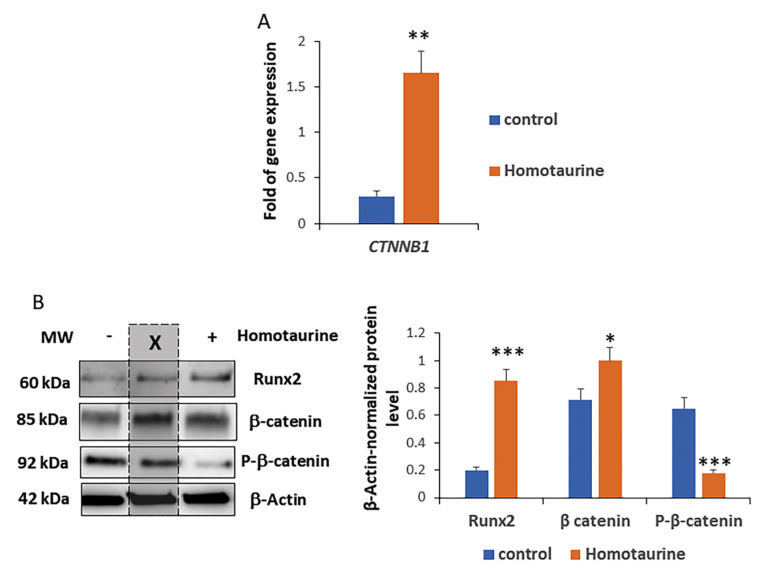
Modulation of Wnt signaling effectors by homotaurine treatment of LRRK2-G2019S-mutated midbrain organoids. (**A**) Gene expression analysis for 4 pooled organoids revealed an increase in *CTNNB1* gene expression levels in response to homotaurine treatment. (**B**) Protein analysis for 4 pooled organoids showed an increase in total β-catenin and RUNX2, along with a decrease in the phosphorylated form of β-catenin, indicating the activation of the Wnt signaling pathway. (* *p* < 0.05; ** *p* < 0.01; *** *p* < 0.005). In the Western blot images, the lanes in the middle marked with the ‘X’ do not contain samples from this study. The original Western blot images are provided in Supplementary Materials S3.

We analyzed the effect of homotaurine treatment on the key effectors of the Wnt signaling pathway, as Wnt activation has been reported to decrease with aging [36,37]. The results showed an increase in the gene expression levels of the *CTNNB1* gene, which encodes β-catenin (Figure 6A), suggesting the activation of the Wnt pathway. Given that the Runx2 gene is a well-established direct target of the canonical WNT signaling pathway mediated by β-catenin [38,39,40], we also analyzed the RUNX2 levels. As shown in Figure 6B, protein analysis revealed elevated levels of total β-catenin and RUNX2, along with a decrease in the phosphorylated form of β-catenin. These findings further indicate that homotaurine treatment may enhance Wnt signaling activity.

## 4. Discussion

The present study investigated the effects of homotaurine on ROS levels, cell viability, and sestrin expression in aging MSCs. It also explored the influence of homotaurine on osteogenesis and angiogenesis in zebrafish models, as well as its potential to decrease ROS levels in a Parkinson’s disease organoid model. Our findings provide valuable insights into the role of homotaurine as a potential therapeutic agent for aging and age-related diseases. Homotaurine, a compound derived from red algae, has been identified as a promising therapeutic agent for neurodegenerative disorders such as Alzheimer’s disease and Parkinson’s disease [41]. Its neuroprotective effects, which include mitigating hippocampal atrophy, enhancing cholinergic transmission, and stabilizing cognitive functions, suggest that it could address the key pathological mechanisms underlying these conditions [41].

The results of this study show a significant increase in ROS levels in MSCs starting from day 3 of culture, which aligns with the well-established relationship between ROS accumulation and cellular aging. Elevated ROS levels are known to induce oxidative stress, contributing to cellular damage and dysfunction in various cell types, including stem cells. The observed increase in ROS levels suggests that aging MSCs may face significant oxidative stress challenges that impact their regenerative capacity.

Interestingly, treatment with homotaurine resulted in a reduction in ROS levels (particularly on day 14 of culture), which was associated with an improvement in cell viability. This finding aligns with previous studies, indicating the potential of homotaurine in modulating oxidative stress [42]. Homotaurine’s ability to lower ROS levels and enhance cell viability may help mitigate the detrimental effects of oxidative stress, promoting the maintenance of MSC function during aging. The sustained positive effects of homotaurine on cell viability observed up to 21 days further support its potential as a therapeutic agent to counteract the aging of stem cells.

Sestrins play a crucial role in maintaining cellular homeostasis and modulating stress responses, particularly under the conditions of oxidative stress [43,44]. The significant increase in sestrin 1 levels following homotaurine treatment suggests that homotaurine may enhance the cellular stress response by activating the mechanisms that protect cells from oxidative damage. The lack of significant changes in sestrin 2 expression implies that homotaurine’s effect on the sestrin family may be selective, further highlighting its potential in regulating specific stress response pathways.

In addition, the reduction in p21 levels following homotaurine treatment suggests that homotaurine may modulate the cell cycle, potentially contributing to enhanced cell survival and function. The unaltered p53 levels indicate that homotaurine may not induce the typical DNA damage response but instead enhances cellular resilience through alternative mechanisms. These findings support the hypothesis that homotaurine’s benefits in aging MSCs may be mediated by the selective modulation of stress-related pathways, such as sestrin 1 and p21 rather than by the broad activation of the cellular damage response.

The impact of homotaurine on skeletal development and angiogenesis was further explored using zebrafish models, providing valuable in vivo insights into its potential effects on aging and regenerative processes. The significant increases in the expression of osteogenic markers such as *ctnnb1*, *Runx2b*, and *Sp7* in both zebrafish larvae and 4-month-old (adult) zebrafish demonstrate homotaurine’s ability to promote osteogenesis. These findings are consistent with the known role of these genes in regulating bone formation, suggesting that homotaurine could support skeletal health and regeneration.

In aged (36-month-old) zebrafish, the enhancement in VEGFR2 expression as a result of homotaurine treatment suggests that homotaurine may improve angiogenesis, which is often impaired during aging. The increased vascular expression of VEGFR2, particularly in the tail region, highlights homotaurine’s potential to promote vascular repair and regeneration, thus improving overall tissue health and regeneration in aging models. These results emphasize homotaurine’s broad regenerative effects, which extend beyond stem cell function to include the promotion of osteogenesis and angiogenesis.

In the context of neurodegenerative diseases such as Parkinson’s disease, the effects of homotaurine were assessed in 3D neural epithelial stem cell (NESC) organoids derived from iPSCs carrying the LRRK2-G2019S mutation.

The lack of impact on colony size suggests that homotaurine does not adversely affect NESC growth during differentiation, which is critical for its therapeutic potential. Additionally, the significant reduction in ROS levels observed in the homotaurine-treated samples suggests that its beneficial effects may be linked to its ability to reduce oxidative stress, a hallmark of Parkinson’s disease. Recent studies also indicate that homotaurine interacts with the Wnt/β-catenin signaling pathway, a critical regulator of neuronal homeostasis that is often disrupted in neurodegenerative diseases [45,46].

Our study extends previous findings on homotaurine’s effects by investigating its potential therapeutic role in NESC 3D colonies derived from Parkinson’s disease models, specifically those harboring the LRRK2-G2019S mutation. This mutation disrupts key cellular pathways, including the Wnt/β-catenin signaling pathway, which is critical for cellular homeostasis, differentiation, and neuroprotection [47,48]. We observed that homotaurine treatment restored β-catenin expression, which is typically dysregulated in carriers of the LRRK2 mutation [49].

In addition to restoring β-catenin, homotaurine significantly reduced the ROS levels in the 3D colonies, addressing a key hallmark of Parkinson’s disease pathology. By mitigating oxidative stress, homotaurine demonstrated its neuroprotective potential, underscoring its promise as a therapeutic candidate for this condition [50].

Thus, our study is the first to evaluate the effects of homotaurine on osteogenic differentiation, highlighting its potential implications for patients with Parkinson’s disease, particularly those with LRRK2 mutations, who often experience compromised bone homeostasis [51]. Reduced bone density and an increased risk of fractures are common in Parkinson’s disease, potentially linked to disruptions in osteogenic differentiation pathways [52]. Our data reveal that homotaurine treatment upregulates RUNX2 expression, a master regulator of osteogenic differentiation, in LRRK2-mutated organoid models. Given that the dysregulation of RUNX2 is observed in both Parkinson’s disease and osteoporosis [53], these findings suggest a shared pathological mechanism that homotaurine may address.

By modulating both β-catenin and RUNX2 expressions, homotaurine demonstrates a multifaceted therapeutic potential. Since the Wnt/β-catenin pathway plays a central role in neuronal and skeletal health, homotaurine’s ability to restore its function represents a dual therapeutic approach. This is particularly relevant for addressing the increased risk of fractures and the associated morbidity in people with Parkinson’s disease.

Although promising, this study has several limitations. The in vitro models, including aging MSCs and Parkinson’s disease organoids, cannot fully replicate the complexity of human physiology, and further validation in mammalian systems is needed. Similarly, while the zebrafish models provided valuable insights into osteogenesis and angiogenesis, the differences between zebrafish and human biology limit direct clinical translation.

Mechanistically, although this study highlights pathways such as Wnt/β-catenin and RUNX2, the interactions between homotaurine’s antioxidant effects and these signaling cascades require deeper exploration. Moreover, this study primarily focused on short- to medium-term effects. Therefore, the long-term safety and efficacy of homotaurine, particularly in chronic conditions such as Parkinson’s disease and osteoporosis, remain to be thoroughly explored. Finally, no clinical trials have yet evaluated homotaurine’s effects on bone density, fracture risk, or neurodegenerative symptoms. Addressing these limitations through advanced preclinical models and clinical studies is essential to confirm its therapeutic potential. However, by demonstrating its impact on oxidative stress, osteogenesis, and angiogenesis in different models, this study paves the way for future investigations into the mechanisms of action and translational potential of homotaurine.

## 5. Conclusions

This study demonstrated that homotaurine reduced ROS levels and improved cell viability in aging MSCs. Similarly, in an in vivo zebrafish model, homotaurine promoted osteogenesis and angiogenesis. These findings highlight the potential of homotaurine to support skeletal and vascular health, particularly under advanced aging conditions. Importantly, homotaurine significantly reduced ROS levels and restored β-catenin expression, thus modulating the Wnt/β-catenin signaling pathway, in a Parkinson’s disease organoid model. While these results are promising, this study has some limitations. The in vitro and zebrafish models, although informative, cannot fully replicate the complexity of human aging, neurodegeneration, or skeletal fragility, and further validations in mammalian systems and clinical studies are needed. Moreover, while this study investigated key pathways such as Wnt/β-catenin and RUNX2, the precise mechanisms underlying their regulation by homotaurine remain to be fully elucidated. Overall, this study provides novel insights into the antioxidant, osteogenic, and angiogenic effects of homotaurine, suggesting that homotaurine represents an innovative therapeutic strategy for neurodegeneration and skeletal fragility. Future investigations will be necessary to further elucidate the role of homotaurine in the interplay between Wnt/β-catenin signaling and RUNX2 regulation in bone and neuronal cells. Additionally, clinical studies should evaluate the impact of homotaurine on bone density and fracture risk in people with Parkinson’s disease carrying LRRK2 mutations.

## Figures and Tables

**Table 1:** Neuronal markers for confocal imaging.

Primary Antibody	Dilution	Origin	Secondary Antibody Fluorophore
TH	1:1000	Cell Signaling (Danvers, MA, USA)	Alexa568
GFAP	1:1000	Cell Signaling (Danvers, MA, USA)	Alexa488
MAP2	1:500	Cell Signaling (Danvers, MA, USA)	Alexa647

## Data Availability

The data presented in this study are available upon request from the corresponding author.

## Supplementary Materials

The supporting information can be downloaded at: https://www.mdpi.com/article/10.3390/antiox14030249/s1, S1: Western Blot images Figure 2A; S2: Western Blot images Figure 2C,D; S3: Western Blot images Figure 6B.

## References

[1] Leyane T.S., Jere S.W., Houreld N.N. (2022). Oxidative stress in ageing and chronic degenerative pathologies: Molecular mechanisms involved in counteracting oxidative stress and chronic inflammation. Int. J. Mol. Sci..

[2] Zuo L., Prather E.R., Stetskiv M., Garrison D.E., Meade J.R., Peace T.I., Zhou T. (2019). Inflammaging and oxidative stress in human diseases: From molecular mechanisms to novel treatments. Int. J. Mol. Sci..

[3] Liguori I., Russo G., Curcio F., Bulli G., Aran L., Della-Morte D., Gargiulo G., Testa G., Cacciatore F., Bonaduce D. (2018). Oxidative stress, aging, and diseases. Clin. Interv. Aging.

[4] Blaser M.C., Aikawa E. (2018). Roles and regulation of extracellular vesicles in cardiovascular mineral metabolism. Front. Cardiovasc. Med..

[5] Nguyen V.V., Witwer K.W., Verhaar M.C., Strunk D., van Balkom B.W. (2020). Functional assays to assess the therapeutic potential of extracellular vesicles. J. Extracell. Vesicles.

[6] Ude A., Ogbodo E., Okeke K. (2022). Stromal Cells and Extracellular Vesicles. Cancer Metastasis-Molecular Mechanism and Clinical Therapy.

[7] Nurkovic J., Volarevic V., Lako M., Armstrong L., Arsenijevic N., Stojkovic M. (2016). Aging of stem and progenitor cells: Mechanisms, impact on therapeutic potential, and rejuvenation. Rejuvenation Res..

[8] Glowacki J., Vokes T. (2016). Osteoporosis and mechanisms of skeletal aging. Advances in Geroscience.

[9] Figueroa C.A., Rosen C.J. (2020). Parkinson’s disease and osteoporosis: Basic and clinical implications. Expert Rev. Endocrinol. Metab..

[10] Invernizzi M., Carda S., Viscontini G.S., Cisari C. (2009). Osteoporosis in Parkinson’s disease. Park. Relat. Disord..

[11] Liu T., Wu H., Li J., Zhu C., Wei J. (2024). Unraveling the Bone–Brain Axis: A New Frontier in Parkinson’s Disease Research. Int. J. Mol. Sci..

[12] Torsney K.M., Noyce A.J., Doherty K.M., Bestwick J.P., Dobson R., Lees A.J. (2014). Bone health in Parkinson’s disease: A systematic review and meta-analysis. J. Neurol. Neurosurg. Psychiatry.

[13] Andrade S., Ramalho M.J., Loureiro J.A., Pereira M.d.C. (2019). Natural compounds for Alzheimer’s disease therapy: A systematic review of preclinical and clinical studies. Int. J. Mol. Sci..

[14] Manzano S., Agüera L., Aguilar M., Olazarán J. (2020). A review on tramiprosate (homotaurine) in Alzheimer’s disease and other neurocognitive disorders. Front. Neurol..

[15] Kelly R.R., Sidles S.J., LaRue A.C. (2020). Effects of neurological disorders on bone health. Front. Psychol..

[16] Zhao Y., Shen L., Ji H.-F. (2013). Osteoporosis risk and bone mineral density levels in patients with Parkinson’s disease: A meta-analysis. Bone.

[17] Davinelli S., Chiosi F., Di Marco R., Costagliola C., Scapagnini G. (2017). Cytoprotective effects of citicoline and homotaurine against glutamate and high glucose neurotoxicity in primary cultured retinal cells. Oxidative Med. Cell. Longev..

[18] Filippelli M., Campagna G., Vito P., Zotti T., Ventre L., Rinaldi M., Bartollino S., Dell’Omo R., Costagliola C. (2021). Anti-inflammatory effect of curcumin, homotaurine, and vitamin D3 on human vitreous in patients with diabetic retinopathy. Front. Neurol..

[19] Kimmel C.B., Ballard W.W., Kimmel S.R., Ullmann B., Schilling T.F. (1995). Stages of embryonic development of the zebrafish. Dev. Dyn..

[20] Whitlock K.E., Westerfield M. (2000). The olfactory placodes of the zebrafish form by convergence of cellular fields at the edge of the neural plate. Development.

[21] Du S.J., Frenkel V., Kindschi G., Zohar Y. (2001). Visualizing normal and defective bone development in zebrafish embryos using the fluorescent chromophore calcein. Dev. Biol..

[22] Reinhardt P., Schmid B., Burbulla L.F., Schöndorf D.C., Wagner L., Glatza M., Höing S., Hargus G., Heck S.A., Dhingra A. (2013). Genetic correction of a LRRK2 mutation in human iPSCs links parkinsonian neurodegeneration to ERK-dependent changes in gene expression. Cell Stem Cell.

[23] Zagare A., Gobin M., Monzel A.S., Schwamborn J.C. (2021). A robust protocol for the generation of human midbrain organoids. STAR Protoc..

[24] Dalle Carbonare L., Bertacco J., Marchetto G., Cheri S., Deiana M., Minoia A., Tiso N., Mottes M., Valenti M.T. (2021). Methylsulfonylmethane enhances MSC chondrogenic commitment and promotes pre-osteoblasts formation. Stem Cell Res. Ther..

[25] Dalle Carbonare L., Gomez Lira M., Minoia A., Bertacco J., Orsi S., Lauriola A., Li Vigni V., Gandini A., Antoniazzi F., Zipeto D. (2023). Expression of FBXW11 in normal and disease-associated osteogenic cells. J. Cell. Mol. Med..

[26] Valenti M.T., Zanatta M., Donatelli L., Viviano G., Cavallini C., Scupoli M.T., Dalle Carbonare L. (2014). Ascorbic acid induces either differentiation or apoptosis in MG-63 osteosarcoma lineage. Anticancer Res..

[27] Sarma S.N., Nagano R., Ohsako S. (2019). Tyroxine hydroxylase-positive neuronal cell population is increased by temporal dioxin exposure at early stage of differentiation from human embryonic stem cells. Int. J. Mol. Sci..

[28] Kim M., Kowalsky A.H., Lee J.H. (2021). Sestrins in physiological stress responses. Annu. Rev. Physiol..

[29] Tarangelo A., Magtanong L., Bieging-Rolett K.T., Li Y., Ye J., Attardi L.D., Dixon S.J. (2018). p53 suppresses metabolic stress-induced ferroptosis in cancer cells. Cell Rep..

[30] Lee I.H., Kawai Y., Fergusson M.M., Rovira I.I., Bishop A.J., Motoyama N., Cao L., Finkel T. (2012). Atg7 modulates p53 activity to regulate cell cycle and survival during metabolic stress. Science.

[31] Lähteenvuo J., Rosenzweig A. (2012). Effects of aging on angiogenesis. Circ. Res..

[32] Vila M. (2019). Neuromelanin, aging, and neuronal vulnerability in Parkinson’s disease. Mov. Disord..

[33] Yang Z., Wang K.K. (2015). Glial fibrillary acidic protein: From intermediate filament assembly and gliosis to neurobiomarker. Trends Neurosci..

[34] Weihe E., Depboylu C., Schütz B., Schäfer M.K.-H., Eiden L.E. (2006). Three types of tyrosine hydroxylase-positive CNS neurons distinguished by dopa decarboxylase and VMAT2 co-expression. Cell. Mol. Neurobiol..

[35] DeGiosio R.A., Grubisha M.J., MacDonald M.L., McKinney B.C., Camacho C.J., Sweet R.A. (2022). More than a marker: Potential pathogenic functions of MAP2. Front. Mol. Neurosci..

[36] Brack A.S., Conboy M.J., Roy S., Lee M., Kuo C.J., Keller C., Rando T.A. (2007). Increased Wnt signaling during aging alters muscle stem cell fate and increases fibrosis. Science.

[37] Palomer E., Buechler J., Salinas P.C. (2019). Wnt signaling deregulation in the aging and Alzheimer’s brain. Front. Cell. Neurosci..

[38] Gaur T., Lengner C.J., Hovhannisyan H., Bhat R.A., Bodine P.V., Komm B.S., Javed A., Van Wijnen A.J., Stein J.L., Stein G.S. (2005). Canonical WNT signaling promotes osteogenesis by directly stimulating Runx2 gene expression. J. Biol. Chem..

[39] Cai T., Sun D., Duan Y., Wen P., Dai C., Yang J., He W. (2016). WNT/β-catenin signaling promotes VSMCs to osteogenic transdifferentiation and calcification through directly modulating Runx2 gene expression. Exp. Cell Res..

[40] McCarthy T.L., Centrella M. (2010). Novel links among Wnt and TGF-β signaling and Runx2. Mol. Endocrinol..

[41] Ricciardi L., De Nigris F., Specchia A., Fasano A. (2015). Homotaurine in Parkinson’s disease. Neurol. Sci..

[42] Lau-Cam C.A. (2020). Protective role of taurine and structurally related compounds against diabetes-induced oxidative stress. Diabetes.

[43] Chen S.-D., Yang J.-L., Lin T.-K., Yang D.-I. (2019). Emerging roles of sestrins in neurodegenerative diseases: Counteracting oxidative stress and beyond. J. Clin. Med..

[44] Dalina A., Kovaleva I., Budanov A. (2018). Sestrins are gatekeepers in the way from stress to aging and disease. Mol. Biol..

[45] Walter J., Bolognin S., Poovathingal S.K., Magni S., Gerard D., Antony P.M., Nickels S.L., Salamanca L., Berger E., Smits L.M. (2021). The Parkinson’s-disease-associated mutation LRRK2-G2019S alters dopaminergic differentiation dynamics via NR2F1. Cell Rep..

[46] Liu J., Xiao Q., Xiao J., Niu C., Li Y., Zhang X., Zhou Z., Shu G., Yin G. (2022). Wnt/β-catenin signalling: Function, biological mechanisms, and therapeutic opportunities. Signal Transduct. Target. Ther..

[47] Toledo E.M., Colombres M., Inestrosa N.C. (2008). Wnt signaling in neuroprotection and stem cell differentiation. Prog. Neurobiol..

[48] Gao J., Liao Y., Qiu M., Shen W. (2021). Wnt/β-catenin signaling in neural stem cell homeostasis and neurological diseases. Neuroscientist.

[49] Anand A.A., Khan M.V.M., Kar D. (2023). The Molecular Basis of Wnt/β-Catenin Signaling Pathways in Neurodegenerative Diseases. Int. J. Cell Biol..

[50] Dorszewska J., Kowalska M., Prendecki M., Piekut T., Kozłowska J., Kozubski W. (2021). Oxidative stress factors in Parkinson’s disease. Neural Regen. Res..

[51] Berwick D.C., Javaheri B., Wetzel A., Hopkinson M., Nixon-Abell J., Grannò S., Pitsillides A.A., Harvey K. (2017). Pathogenic LRRK2 variants are gain-of-function mutations that enhance LRRK2-mediated repression of β-catenin signaling. Mol. Neurodegener..

[52] Xiong L., Pan J.-X., Guo H.-h., Mei L., Xiong W.-C. (2021). Parkinson’s in the bone. Cell Biosci..

[53] Minoia A., Dalle Carbonare L., Schwamborn J.C., Bolognin S., Valenti M.T. (2022). Bone tissue and the nervous system: What do they have in common?. Cells.

