# Supplementary figures and images for: Antioxidant, Osteogenic, and Neuroprotective Effects of Homotaurine in Aging and Parkinson’s Disease Models
Source: Antioxidants (Basel). 2025 Feb 21;14(3):249. doi: 10.3390/antiox14030249 (PMC11939455; doi:10.3390/antiox14030249)

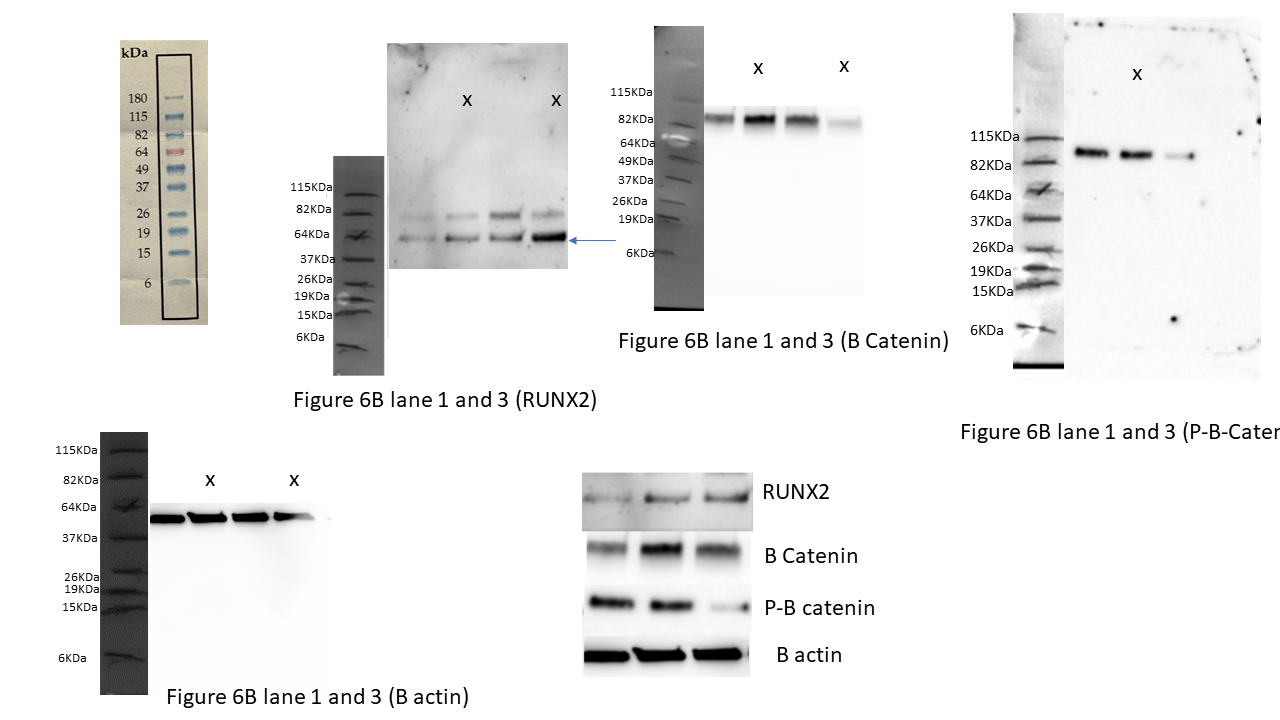

Supplement: Supplementary file 1 [file antioxidants-14-00249-s001.zip › Supplementary Material 3.tif]

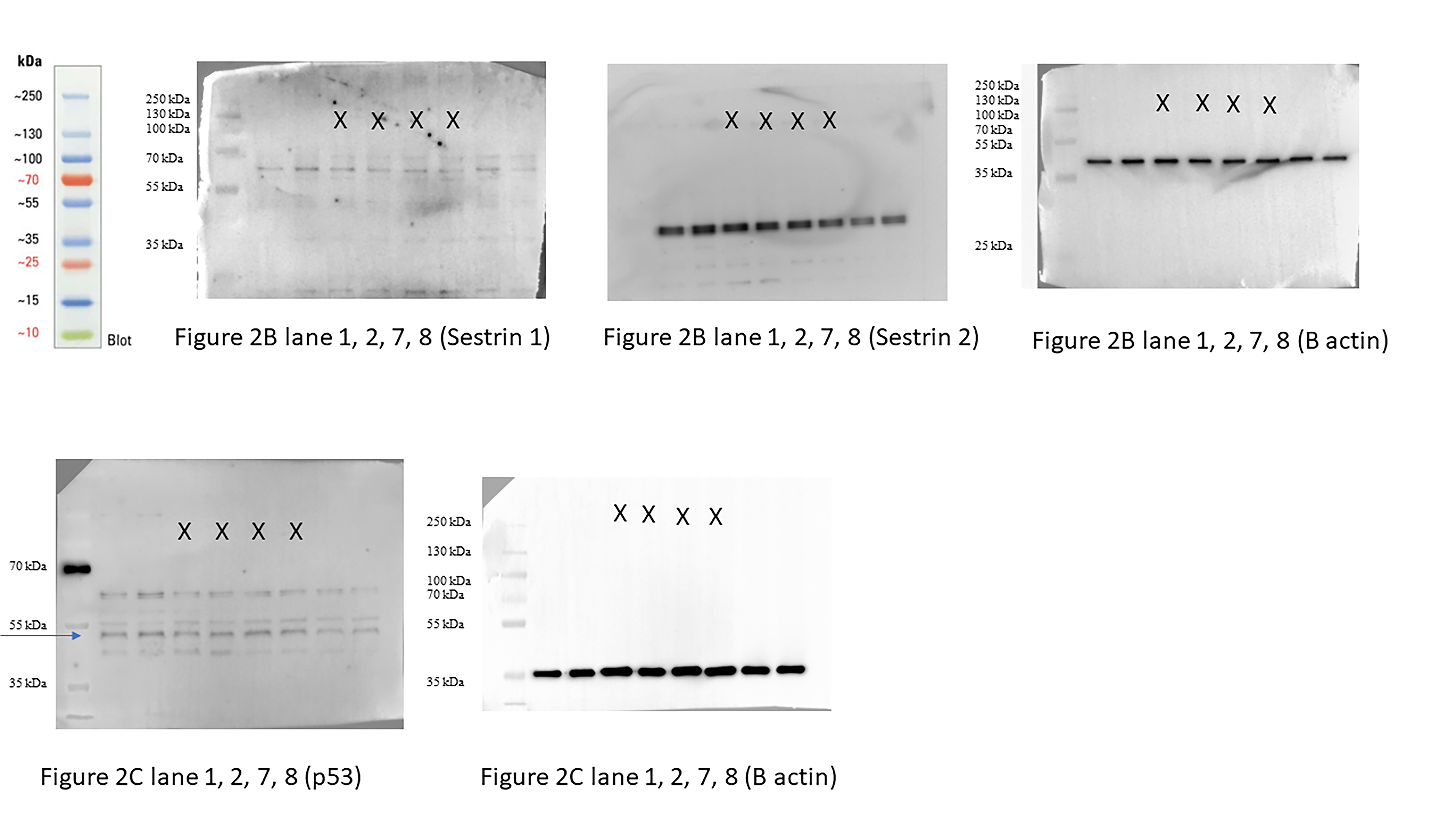

Supplement: Supplementary file 1 [file antioxidants-14-00249-s001.zip › Supplementary materials 1.tif]

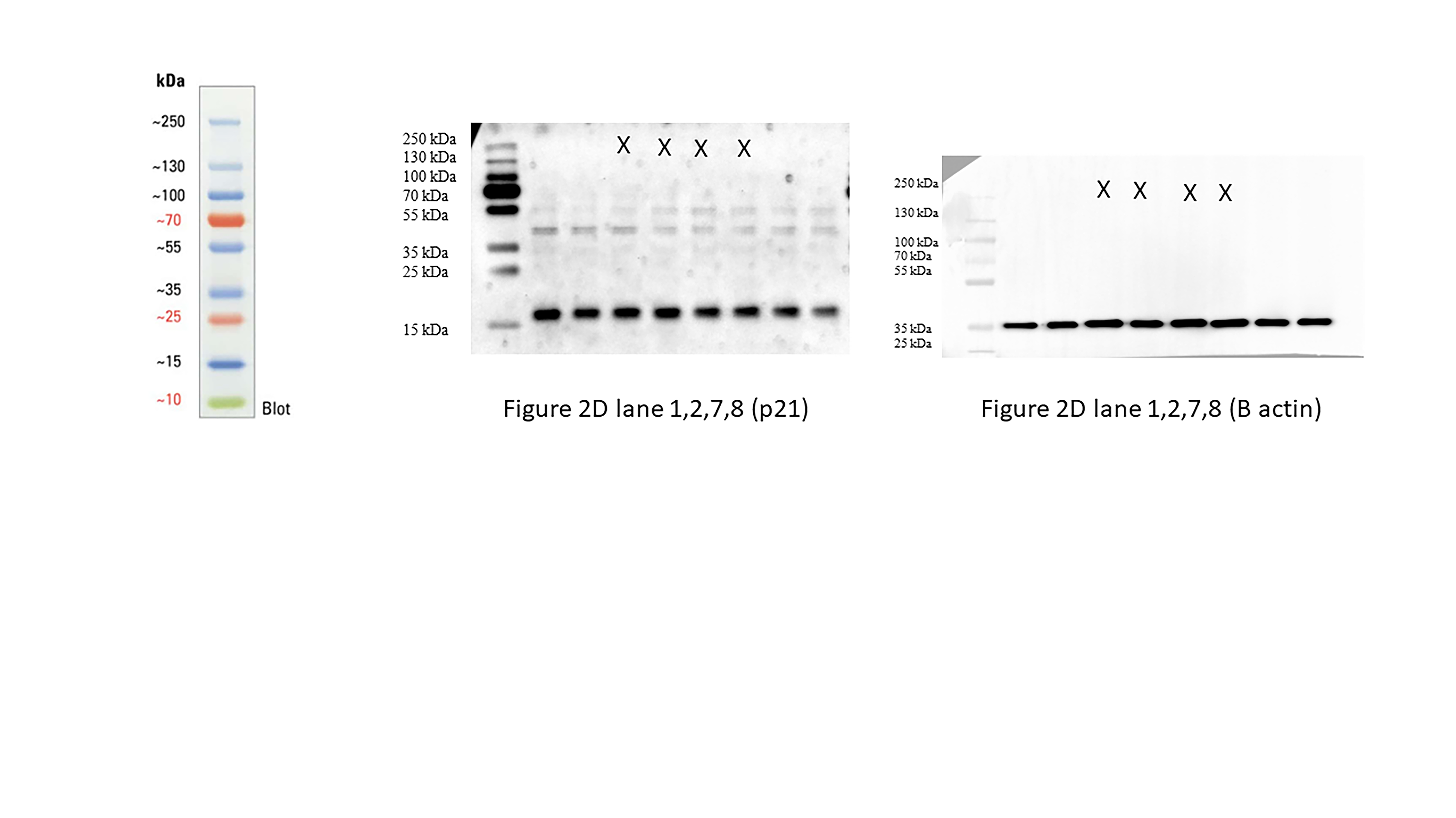

Supplement: Supplementary file 1 [file antioxidants-14-00249-s001.zip › Supplementary materials 2.tif]
